# Immunogenicity Assessment of Rift Valley Fever Virus Virus-Like Particles in BALB/c Mice

**DOI:** 10.3389/fvets.2020.00062

**Published:** 2020-02-13

**Authors:** Yuetao Li, Li Han, Yongkun Zhao, Xuexing Zheng, Hualei Wang, Weiwei Gai, Hongli Jin, Guohua Li, Qi Wang, Na Feng, Yuwei Gao, Songtao Yang, Xianzhu Xia

**Affiliations:** ^1^College of Animal Science and Veterinary Medicine, Henan Institute of Science and Technology, Xinxiang, China; ^2^Institute of Military Veterinary Medicine, Academy of Military Medical Science, Changchun, China; ^3^College of Animal Science and Technology, Shihezi University, Shihezi, China; ^4^School of Public Health, Shandong University, Jinan, China; ^5^Key Laboratory of Zoonosis Research, Ministry of Education, College of Veterinary Medicine, Jilin University, Changchun, China; ^6^Nano Innovation Institute, Inner Mongolia University for the Nationalities, Tongliao, China

**Keywords:** rift valley fever, vaccine, virus-like particle, immunogenicity, cytokine

## Abstract

Rift Valley fever (RVF) is an acute, febrile zoonotic disease that is caused by the RVF virus (RVFV) and is spread by arthropod vectors. Virus-like particle (VLP) vaccines, which have the advantages of strong immunogenicity and safety, play an important role in the prevention of this disease. VLPs for RVFV were successfully prepared by our research group using a baculovirus-insect cell expression system. To study the immunogenicity of these RVFV VLPs, a correct 3rd or 4th generation recombinant baculovirus, rBac-N-G, was identified and used to infect Sf9 cells, which were cultured in suspension at a large scale. Subsequently, cell debris was removed by centrifugation, and the VLPs were concentrated by ultracentrifugation and purified using a sucrose gradient, after which they were used to immunize BALB/c mice by intramuscular injection. The results showed that the RVFV VLPs prepared by our research group could effectively induce mice to produce RVFV neutralizing antibodies and that the prepared VLPs could stimulate mouse spleen cells to produce high levels of the cytokines IL-4 and IFN-γ. Moreover, the proportion of lymphocytes producing IL-4 and IFN-γ in the spleen of mice immunized with RVFV VLPs was significantly increased. Therefore, the RVFV VLPs prepared in this study had strong immunogenicity and could effectively activate humoral and cellular immunity in mice. This study lays a solid foundation for the development of RVFV VLP vaccine candidates and promotes the healthy development of animal husbandry and human public health.

## Introduction

Rift Valley fever (RVF) is a zoonotic disease caused by Rift Valley fever virus (RVFV) that is prevalent in the Arabian Peninsula, African continent and several islands in the southeast Indian Ocean (1, 2). In these areas, RVF has repeatedly erupted among animals and humans (3). Domestic ruminants, especially sheep, are most susceptible to RVFV, and infection in pregnant sheep results in abortion in almost 100% of cases and in nearly 100% mortality in newborn lambs. Although cattle, goats, and wild ruminants are slightly less sensitive to this virus, these animals are also at considerable risk (3). RVFV has been isolated from more than 30 species of mosquitoes, several of which are distributed globally. These features explain why RVFV is one of the most serious arboviruses that threatens human and animal health (2). RVF disease is classified as a Class A disease by the World Organization for Animal Health (OIE) and has been included in the list of diseases that require statutory reporting (4, 5). Though, RVFV is a single-stranded RNA virus (5), it is very stable. Reports, showed that the genetic diversity of RVFV is quite limited, with maximum pairwise differences for the S segment and partial M segments of 4 and 5.4% for the nucleotide sequence and 1 and 2.8% for the amino acid sequence (6).

The US Centers for Disease Control and Prevention (CDC) and the US Department of Agriculture (USDA) list RVFV as a Class A pathogen (5). China lists RVF as one of the 13 exotic animal diseases that require focused prevention in *The National Medium- and Long-term Plan for Animal Disease Control (2012–2020)*. Due to its significant biological impact, this disease must be prevented and controlled.

Laboratory studies have shown that vaccination is the main means of preventing and controlling RVF (5, 7). No specific anti-RVFV drugs are available on the market, nor are there any commercial RVF vaccines. In recent years, virus-like particle (VLP) vaccines, which have the advantages of strong immunogenicity and safety, have shown great potential for novel vaccine development.

VLPs display epitopes in a form close to that of natural viruses, thus rendering them not only highly immunogenic but also easily acquirable by specialized antigen-presenting cells (APCs), i.e., dendritic cells (DCs) (8). VLPs can bind to Ig receptors on B lymphocytes, which activates them and leads to the recruitment of DCs via increased co-stimulatory molecule expression and cytokine production (8). Upon being processed, VLPs are presented by major histocompatibility complex (MHC) class II molecules, thereby stimulating CD4^+^ T cells; VLPs are recognized and presented to CD8^+^ T cells by MHC class I molecules via cross-presentation.

In a previous study, our research group successfully developed a method for the preparation of RVFV VLPs based on an insect baculovirus system (9). In this study, large quantities of high quality RVFV VLPs were obtained using large-scale preparation, concentration, and purification methods, and the prepared VLPs were used to perform mouse immunization studies. The immunogenicity of the RVFV VLPs was examined to determine whether they have the potential to be used to develop a novel RVF vaccine, thereby laying the foundation for the development of a VLP-based RVF vaccine.

## Materials and Methods

### Viruses and Cells

The recombinant baculovirus rBac-N-G, which contains the RVFV Gn, Gc and N proteins, was constructed in a previous study (9). A suspension of Sf9 insect cells (*Spodoptera frugiperda* Sf9 cells) was preserved by the Laboratory of Animal Virology and Special Animal Lemology at the Military Veterinary Research Institute in China; these cells were cultivated per a previously described method (9).

### Experimental Animals

Female, 6–8 weeks-old BALB/c mice with a body weight of 15–16 g were purchased from Changchun Yisi Experimental Animal Co., Ltd., China. The mice were maintained as our previous study (10). All animal feeding was in compliance with animal welfare regulations.

### Preparation of the VLPs

The recombinant baculovirus rBac-N-G, upon being identified as correct, was used to infect suspended Sf9 insect cells, which were cultured in suspension at 120 rpm and 27°C for ~96 h. The cells were then collected and centrifuged at 7,000 rpm and 4°C for 30 min to remove cell debris. The collected culture supernatant was concentrated via ultracentrifugation at 4°C and 35,000 rpm for 2 h; the pellet was resuspended in PBS and dissolved overnight at 4°C. Sucrose solutions of 60, 40, and 20% were prepared in advance and filtered individually with 0.22-μm filters. Subsequently, the 60, 40, and 20% sucrose solutions were, in turn, gently added to a special ultracentrifuge tube. In doing so, a 20–40–60% sucrose gradient was formed from top to bottom in the tube. The PBS resuspended liquid was slowly added to the upper surface of the sucrose gradient (20–40–60% (w/v), which was then centrifuged at 30,000 rpm at 4°C for 120 min. Next, the white matter between the 40 and 60% sucrose liquid levels was collected and added to an ultracentrifuge tube with PBS. The resultant solution was centrifuged at 30,000 rpm for 1.5 h, after which the supernatant was discarded, and the pellet was resuspended in PBS and dissolved overnight at 4°C. The solution concentration was determined using a BCA protein assay kit (Beyotime, China), and other methods were used for verification. The final concentration was adjusted to 1 mg/ml, and the solution was aliquoted and stored at −80°C for further use.

### VLP Immunization of Mice

**Figure 1:**
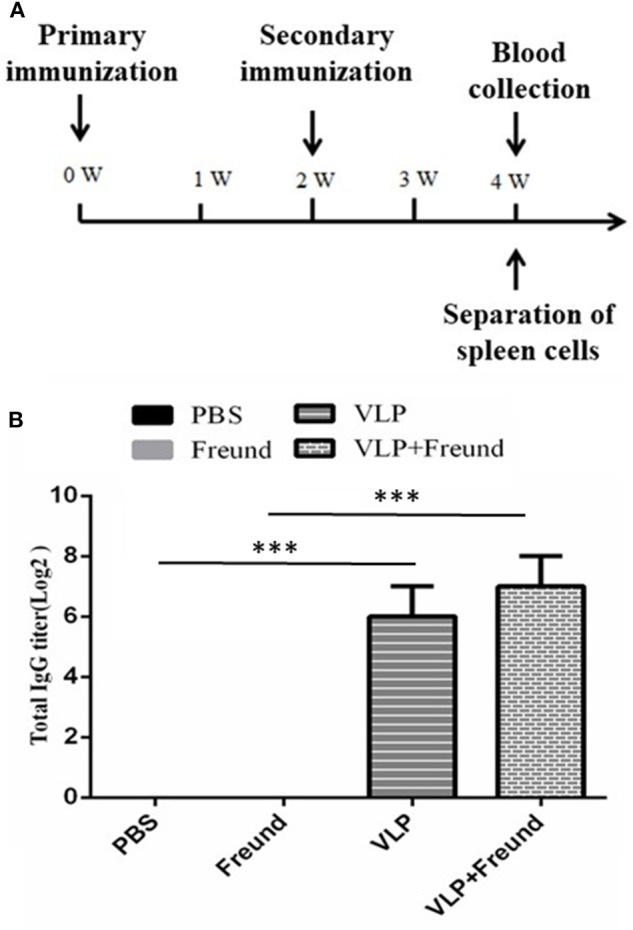
Analysis results of serum IgG levels after immunization with RVFV VLPs in mice. **(A)** Mouse immunization protocol; W, week. **(B)** Serum IgG levels in the immunized mice. Blood was collected from the mice at day 14 after the second immunization, and the serum IgG titers were measured by ELISA. The data were expressed as the mean ± SD (*n* = 10, ***p < 0.001).

A wide host range can be experimentally infected with RVFV, but as mice can be infected by various means, they are a good animal model for studying the pathogenesis of RVFV infection in large mammals. In this experiment, mice were immunized with the purified RVFV VLPs. The mice were randomly divided into 4 groups of 10, with the first group being the PBS group, the second group being the Freund's adjuvant group, the third group being the VLP group and the fourth group being the VLP + Freund's adjuvant group. Freund's complete adjuvant and Freund's incomplete adjuvant were used for the first and second immunization, respectively, with an immunization dose of 100 μl/mouse via hind limb intramuscular injection. Group one was intramuscularly injected with PBS, group two with Freund's adjuvant (PBS and Freund were mixed at an equal volume ratio of 1:1), group three with VLPs (15 μg VLPs/mouse) and group four with VLPs + Freund's adjuvant (15 μg VLPs/mouse). Blood was collected from the mice at 2 weeks after the second immunization, and immunity-related indexes were determined. The design of the immunization protocol is shown in Figure 1A.

### Determination of Immune Indexes in Mice

To measure IgG-specific antibodies, the mice were euthanized at 4 weeks after the primary immunization, and blood was collected. The blood samples were kept at room temperature for 2 h and then centrifuged at 3,000 rpm for 10 min to obtain serum. An indirect ELISA method was used to determine the RVFV antibody titers in the mouse sera after the second immunization. The Gn protein was used for coating at a concentration of 10 μg/ml, with incubation overnight at 4°C; the plates were then blocked with 1% BSA for 120 min. The serum samples were used as the primary antibody, and the plates were incubated at 37°C for 120 min after their addition. Goat anti-mouse IgG was used as the secondary antibody, with incubation at 37°C for 60 min, after which the tetra methyl benzidine (TMB) substrate was added and incubated at room temperature for 15 min. The stop solution was added, and the OD_450nm_ value of each sample was determined using a microplate reader (11).

A serum neutralization test was carried out as follows. A previously, constructed RVFV pseudovirus (with an EGFP marker) and neutralization assay (11) was used to determine antibody-neutralizing activity in serum from immunized mice that was collected at day 14 after the second immunization. Antibody-neutralizing activity was calculated using the following formula: antibody-neutralizing activity = [(number of green fluorescent cells in the control group—number of green fluorescent cells in the test group)/number of green fluorescent cells in the control group ×100%] (12).

IFN-γ and IL-4 levels were determined using an IFN-γ and IL-4 ELISpot assay kit (AID, Germany). At day 14 after the second immunization, spleens were collected from the mice with and without VLPs immunization to isolate spleen cells. All procedures were performed in a biosafety cabinet.

Intracellular cytokine staining (ICS) analysis was conducted at day 14 after the second immunization. The mouse spleens with and without VLPs immunization were collected, and spleen cells were isolated for ICS of IL-4 and IFN-γ in CD8^+^ or CD4^+^ T cells. Flow cytometry was used to determine cellular fluorescence.

### Statistical Analysis

All data were expressed as the mean ± SD, and statistical analyses were performed using GraphPad Prism 5 software (GraphPad Software Inc., San Diego, CA, USA). Student's *t*-test was performed to evaluate differences. Significant difference was set at *P* < 0.05.

## Results

### Determination of IgG Levels After Immunizing Mice With RVFV VLPs

The RVFV VLPs immunizations were conducted according to the Figure 1A. At day 14 after the second immunization, the serum IgG titers were determined. The results, showed that the IgG antibodies against the RVFV VLPs were not produced in either the first group (the PBS group) or the second group (the Freund's adjuvant group) of mice. The highest titer, 1:2^7^, was produced in the fourth group (the VLPs + Freund's adjuvant group) of mice. The third group (the VLP group) mice also produced a high serum titer, 1:2^6^, in the absence of any adjuvant. The results were shown in Figure 1B. The results showed that RVFV VLPs could induce mice to produce a strong humoral immune response in mouse model.

### Analysis of Serum Neutralizing Antibodies in Mice

**Figure 2:**
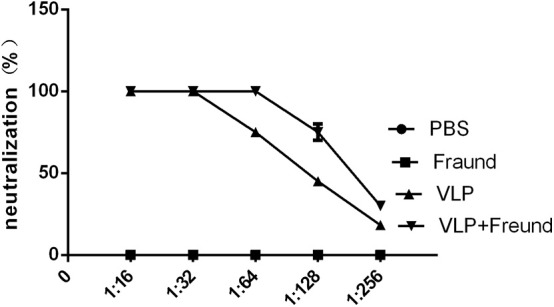
Determination of antibody neutralizing activity in immunized mice. Serum samples were collected and doubly diluted, and the neutralizing activity was determined using an RVFV pseudovirus previously, prepared by our laboratory.

The sera prepared from the blood samples taken from the mice on day 14 after the second immunization were doubly diluted, and neutralizing activity was determined using an HIV-based lentiviral-packaged RVFV pseudovirus (with an EGFP marker) (13) prepared by and stored in our laboratory. The experimental results, are shown in Figure 2; no neutralizing activity was detected in sera from mice in the first group (the PBS group) or second group (the Freund's adjuvant group). Sera from mice in the fourth group (the VLPs + Freund's adjuvant group) demonstrated 70% neutralization activity at a dilution of 1:2^7^ and 100% neutralization activity at a dilution of 1:2^6^. In sera from mice in the third group (the VLP group, which did not include an adjuvant), 100% neutralization activity at a dilution of 1:2^5^ and 60% neutralization activity at a dilution of 1:2^6^ were observed. These results showed that VLP-based experimental vaccines have high immunogenicity in mouse models and can induce high levels of neutralizing antibodies even in the absence of adjuvants.

### IFN-γ and IL-4 Secretion in Spleen Cells From Mice Immunized With RVFV VLPs

**Figure 3:**
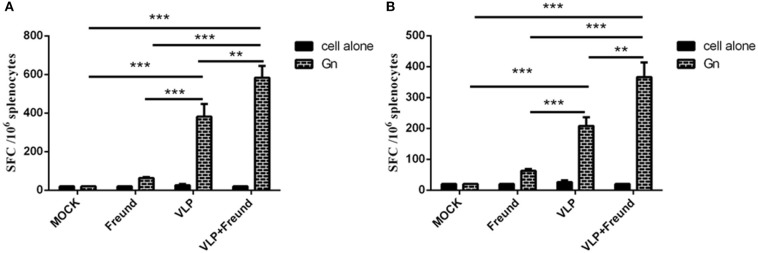
Enzyme-linked immune spot assay for IL-4 **(A)** and IFN-γ **(B)** production by spleen lymphocytes from mice at day 14 after the second immunization. After the spleen cells were isolated, the IL-4 and IFN-γ levels were determined using an ELISpot assay kit; all procedures were performed in a biosafety cabinet (**p < 0.01, ***p < 0.001).

To determine whether the RVFV VLPs can induce cytokine production in lymphocytes, spleen lymphocytes were isolated at 2 weeks after the booster immunization (week 4). Subsequently, the spot-forming cells (SFCs) produced by the lymphocytes with partial Gn protein stimulation were detected to analyze the ability of the lymphocytes to produce cytokines such as IFN-γ and IL-4. The results are shown in Figure 3. Under stimulation with purified Gn protein, lymphocytes from mice in the fourth group (the VLPs + Freund's adjuvant group) produced the highest levels of IFN-γ and IL-4 compared to those in the other three groups. Lymphocytes from mice in the third group (the VLP group) also produced high levels of cytokines in the absence of Freund's adjuvant. These results showed that the RVFV VLPs induced antigen-specific cellular immune responses in the mice.

### RVFV VLP-Induced Responses in CD4^+^ and CD8a^+^ T Lymphocytes

**Figure 4:**
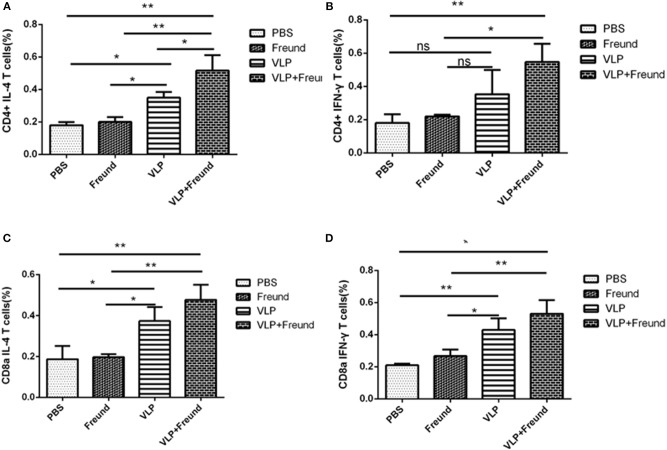
RVFV VLP-induced responses in CD4^+^ and CD8a^+^ T lymphocytes. **(A)** IL-4 levels in CD4^+^ T cells determined by intracellular cytokine staining (ICS). **(B)** IFN-γ levels in CD4^+^ T cells determined by ICS. **(C)** IL-4 levels in CD8^+^ T cells determined by ICS, and **(D)** IFN-γ levels in CD9^+^ T cells determined by ICS (*p < 0.05, **p < 0.01).

To effectively assess the intensity of the cellular immune response and to further evaluate the activation of T lymphocytes induced by the RVFV VLPs in mice, spleen cells were isolated at 2 weeks after the booster immunization (week 4), and ICS was used to analyze the synthesis and secretion of IFN-γ and IL-4 in CD4^+^ and CD8^+^ T cells. The difference between the percentage of cells that were both cytokine-positive and lymphocyte-positive when Gn protein was added and that of cells that were both cytokine-positive and lymphocyte-positive in the absence of Gn protein were graphed for each cytokine, and the results are shown in Figure 4.

The levels of the two cytokines, IL-4 and IFN-γ, produced by the T lymphocytes (CD4^+^) isolated from mice in the fourth group (the VLPs + Freund's adjuvant group) were significantly higher than those in the other three groups (*P* < 0.05; Figures 4A,B). T lymphocytes (CD4^+^) from mice in the fourth group (the VLPs + Freund's adjuvant group) produced a significantly higher level of IFN-γ than that of the third group (the VLP group) (*P* < 0.05; Figure 4B), but their IL-4 production level was only slightly increased (Figure 4A). Therefore, these results indicated that the VLPs with Freund's adjuvant could significantly increase the immune response produced by antigen-specific CD4^+^ T cells.

Since T lymphocytes (CD8^+^) play an important role in the fight against viral infection, ICS was used to assess IL-4 and IFN-γ cytokine production in T lymphocytes (CD8^+^) (Figures 4C,D). IFN-γ cytokine production by spleen T lymphocytes (CD8^+^) from mice in the fourth group (the VLPs + Freund's adjuvant group) was significantly higher than that in the other three groups (*P* < 0.01). Moreover, for lymphocytes from mice in the third group (the VLP group) in which the adjuvant was absent, secreted IFN-γ cytokine production by T lymphocytes (CD8^+^) was also high. IL-4 secretion by T lymphocytes (CD8^+^) was similar to that of IFN-γ. The secretion of IL-4 and IFN-γ by T lymphocytes (CD8^+^) from mice in the first group (the PBS group) and second group (the Freund's adjuvant group) was extremely low.

## Discussion

RVF can be transmitted by mosquito bites and by contact with contaminated materials, and it has a mortality rate of approximately 1%. However, in recent epidemics, the mortality rate has increased significantly (14). At present, no specific anti-RVFV drugs are available on the market, nor are there any commercial RVFV vaccines for human use. Currently, the primary means of preventing RVFV is vaccination. There are three main vaccine types: recombinant live vector vaccines, inactivated vaccines, and live attenuated vaccines. These vaccines each have their own shortcomings, limiting their application and effectiveness during RVFV outbreaks.

VLPs have a polyprotein structure that mimics that of their natural virus in morphology and structure and is free of viral genetic material, thus making them safe and expensive candidate vaccines. At present, VLP-based commercially available preventive vaccines have been developed worldwide (15), including Glaxo Smith Kline's Engerix (for hepatitis B virus) and Cervarix (for human papillomavirus) as well as Merck's Recombivax HB (for hepatitis B virus) and Gardasil (for human papillomavirus) (16). In 2011, the Hecoin vaccine for hepatitis E virus (HEV) that was developed by Innovax Biotech of Xiamen, China, was approved for use in China. In the veterinary field, a vaccine for porcine circovirus type 2 (PCV-2) developed using VLPs has been approved for marketing (Intervet International Ltd., the Netherlands) (17). VLPs can effectively activate APCs and B lymphocytes by virtue of their size, potential and other properties (18, 19). Moreover, VLPs can be processed and presented through two pathways, the MHC-II and MHC-I molecular pathways, to induce strong humoral and cellular immune responses (20–22), which allows DCs to efficiently induce cellular T lymphocyte responses in the absence of viral replication (23). Therefore, some VLPs can activate responses in both helper T cells and cytotoxic T lymphocytes. VLPs can also directly induce DC maturation, thereby positively regulating co-stimulatory molecules and cytokines to activate T lymphocytes (24, 25).

In this study, a previously constructed recombinant baculovirus (9), rBac-N-G, which contains the RVFV Gn, Gc, and N proteins, was used to infect Sf9 insect cells to express RVFV VLPs. Although the virion cannot replicate, it can be adsorbed to cell receptors, in a manner similar to that of cells infected with RVFV. VLPs have certain advantages compared with soluble proteins, including adequate antigen stability and immunogenicity. VLPs are first adsorbed to cellular receptors, after which the virion envelope proteins are digested by lysosomes and the antigen is presented by MHC (26). The rBac-N-G recombinant baculovirus was used to infect the Sf9 insect cells, which were cultured at large scale. The RVFV VLPs were concentrated and purified and then used to immunize mice. The experimental results showed that the RVFV VLPs induced mice to produce high-specificity immunoglobulins and neutralizing antibodies.

RVFV VLPs have been generated by several other researchers (27–29), and the animal model experiments confirmed its effectiveness in the induction of virus neutralizing antibody (28, 29). However, the cellular immune response induced by the RVFV VLPs has not been studied. In this study, a cellular immune response was triggered by the RVFV VLPs in the RVFV VLPs immunized mice. Result showed that the VLPs significantly increased/stimulated the secretion of high levels of the cytokines IL-4 and IFN-γ; they also significantly increased the proportion of CD8^+^ and CD4^+^ T lymphocytes secreting IL-4 and IFN-γ in the mouse spleen cells. IFN-γ is a Th1-type cytokine, and IL-4 a Th2-type cytokine, indicating that the VLPs alone can induce a balanced Th1/Th2 immune response in mice. Hence, the prepared RVFV VLPs could induce a strong immune response in small animals. This study showed that VLP-based experimental vaccines (strains) have good immunogenicity in mouse models and can induce high levels of neutralizing antibodies even in the absence of adjuvants.

However, many tasks must be completed to prepare a vaccine with RVFV VLPs using the Bac-to-Bac system. The immunogenicity of the VLPs would be further improved and the production costs would be reduced, thereby laying the foundation for the development of vaccines using RVFV VLPs. In addition, there are some limitations in this study. For example, the virus challenge experiment after vaccine immunization is lacking due to the limited experiment conditions. Further studies are needed for evaluating the vaccine efficiency using animal models.

## Data Availability Statement

All datasets generated for this study are included in the article/supplementary material.

## Ethics Statement

All the animal studies were conducted according to the Guidelines for the Care and Use of Laboratory Animals (No. 55 issued by the Ministry of Health, People's Republic of China, on January 25, 1998), and all efforts were made to minimize suffering. The study protocol was approved by the Animal Ethics Committee of the Institute of Military Veterinary Medicine at the Academy of Military Medical Science.

## Author Contributions

YL, SY, and XX designed the experiment. YL, LH, and YZ performed the experiments. XZ, HW, WG, HJ, and GL helped with the experiments. QW, NF, and YG analyzed the experimental results and developed analysis tools. YL wrote the paper.

### Conflict of Interest

The authors declare that the research was conducted in the absence of any commercial or financial relationships that could be construed as a potential conflict of interest.
